# 

**Published:** 1920-12

**Authors:** 


					CONTENTS. Page
^"edicine and Surgery.
By L. E. V. Every-Clayton, M.D. Lond.
193
Diagnosis of Torsion of the Great Omentum, illustrated by
two Case-Histories.
By James Swain, C.B., M.S., M.D. Lond.,
F.R.C.S.
Clinical Significance of Adsorption Phenomena.
By
Oliver C. M. Davis, M.D. Bristol
205
uberculosis and Consumption in Relation to Public Health.
oy D. S. Davies, M.D. Lond , LL.D., D.P.H.
^?views of Books
224
Editorial
Notes
229
Th
e Library of the Bristol Medico-Chirurgical Society   234
eetings of Societies
236
^?cal Medical Note
242

				

